# Retraction Note to: EV71 virus reduces Nrf2 activation to promote production of reactive oxygen species in infected cells

**DOI:** 10.1186/s13099-023-00582-9

**Published:** 2023-11-28

**Authors:** Zhenzi Bai, Xiaonan Zhao, Chenghua Li, Chuanlun Sheng, Hongyan Li

**Affiliations:** Infectious Department, China?Japan Union Hospital, Jilin University, No.126, Xiantai Street, Economic Development Zone, Changchun, 130033 Jilin China

**Retraction to: Bai et al. Gut Pathog (2020) 12:22**

10.1186/s13099-020-00361-w.

The editors have retracted this article. After publication, concerns were raised regarding similarities of western blots, fluorescence images and flow cytometry plots presented in Figs. 1, 2, 3, 4, 5, 6, 7 and 9 with other papers that were either published earlier or under consideration within a similar time frame [1–6]. Additionally, some of the flow cytometry plots appear to be duplicated in Figs. 6 and 9, and a number of western blots appear highly similar in Figs. 3, 5 and 7.

The authors have confirmed that incorrect images were used in this article, and the raw data are no longer available.

The editors therefore no longer have confidence in the presented data.

None of the authors have responded to any correspondence from the editor or publisher about this retraction notice.
